# ‘It breaks my heart’: Healthcare practitioners’ caring for families with epidermolysis bullosa

**DOI:** 10.4102/hsag.v28i0.2355

**Published:** 2023-10-24

**Authors:** Antoinette V. Chateau, Colleen Aldous, Ncoza Dlova, David Blackbeard

**Affiliations:** 1 Department of Dermatology, Grey’s Hospital, Pietermaritzburg, South Africa; 2 Department of Dermatology, Faculty of Health Science, University of KwaZulu-Natal, Durban, South Africa; 3 School of Clinical Medicine, Faculty of Health Science, University of KwaZulu-Natal, Durban, South Africa; 4 Department of Psychology, Grey’s Hospital, Pietermaritzburg, South Africa; 5 Department of Psychiatry, Faculty of Health Science, University of KwaZulu-Natal, Durban, South Africa

**Keywords:** epidermolysis bullosa, genetic skin disease, rare disease, healthcare practitioners, nurses, doctors, impact, perceptions, needs, interpretive phenomenological analysis

## Abstract

**Background:**

Epidermolysis bullosa (EB) is a painful genodermatosis presenting with skin fragility and blisters. There is no cure; the prognosis is guarded and depends on the subtype of the disease. Managing these patients can be emotionally challenging for healthcare practitioners.

**Aim:**

To determine the perceptions, impact, and needs of healthcare practitioners (HCP) caring for patients and their families with EB.

**Setting:**

Nelson Mandela School of Medicine, Durban and Grey’s Hospital, Pietermaritzburg, KwaZulu-Natal.

**Methods:**

The study was guided by interpretative phenomenological analysis. Individual in-depth interviews were conducted with 10 healthcare practitioners. Guba’s trustworthiness framework was used to ensure rigour.

**Results:**

Six global themes were identified, each related primarily to the perceptions, impact, and needs of healthcare practitioners. The experiences and perceptions of healthcare practitioners were that caring for patients with an incurable disease such as EB could negatively impact healthcare practitioners. There were divergent views among the disciplines of HCPs regarding the extent of care in a resource-limited environment. This resulted in negative emotions, ethical concerns, and a need for continued medical education and the application of coping strategies. Healthcare practitioners observed that patients and their families were vulnerable, requiring comprehensive biopsychosocial care.

**Conclusion:**

Healthcare practitioners should be aware of their emotional challenges, seek support where necessary, and use effective coping strategies and self-care.

**Contribution:**

The concerns and needs of healthcare practitioners are highlighted and interventional strategies to assist healthcare practitioners are suggested which will ultimately improve patient care.

## Introduction

Epidermolysis bullosa (EB) is a rare genodermatosis that presents with skin fragility. There are four main types of EB, namely EB simplex, junctional EB, dystrophic EB and Kindler syndrome. Pain is a common symptom in all the subtypes of EB (Has et al. 2020). The condition ranges from mild blister formation to severe disease involving a large surface area, which can result in death. Complications include infection, ocular, gastrointestinal, cardiac, renal, and skeletal anomalies, and cutaneous malignancies (Fine & Mellerio 2009a, 2009b). Treatment aims to control pain, prevent new blister formation, and prevent and manage complications. There is no cure for this condition (El Hachem et al. 2014).

Epidermolysis bullosa impacts the quality of life of the patient and their families. In older patients, it can affect day-to-day living, schooling, interpersonal functioning, relationships, family planning, and job opportunities of the patient (Dures et al. 2011).

Researchers have found that parents of children with EB are concerned about healthcare practitioners’ limited knowledge about the condition, which parents also perceive leads to delays in referrals to specialist centres (Kearney, Donohoe & Mcauliffe 2020; Van Scheppingen et al. 2008; Wu, Sun & Lee 2020; Yuen, Duipmans & Jonkman 2012). Parents reported feeling under-equipped to care for their child’s condition, resulting in incorrect dressings applied to fragile and painful wounds (Van Scheppingen et al. 2008; Yuen et al. 2012). Parents reported feeling hesitant to seek medical assistance because healthcare practitioners did not validate their views (Kearney et al. 2020). They also experienced having to re-explain the condition each time they consulted a new healthcare practitioner (Kearney et al. 2020). Some parents perceived healthcare practitioners as insensitive or dismissive (Dures et al. 2011). Parents expressed a need for rapid referral to expert opinion, care, education about the condition, and support. Parents appreciated the honesty of healthcare practitioners regarding the child’s prognosis and wanted to be involved in decision-making and end-of-life care (Yuen et al. 2012).

A good practitioner-patient relationship is essential for the holistic care of the patient and the family. Many healthcare practitioners encourage a collaborative role with their patients, yet some might engage less well with assertive parents or patients (Budych, Helms & Schultz 2012). Few specialist EB centres globally have trained professionals to care for these patients and their families (Dures et al. 2010). The scarcity of skilled practitioners and units is likely to be more challenging in a resource-limited environment such as South Africa. The advanced care of rare, severe conditions such as EB gives rise to ethical debates and conflicts, such as whether or not it is right to treat, especially in a resource-limited environment, or other questions of potential moral injury (Boesen et al. 2016; Norup 1999).

In a study by Dures et al. (2010), healthcare practitioners expressed feeling demoralised, powerless, inadequate, self-critical and guilty about being unable to cure the patient and alleviate distress. Recently, the coronavirus disease 2019 (COVID-19) pandemic has also been challenging for parents and healthcare practitioners concerning healthcare and physical interaction (Murrell et al. 2020; Vente 2021). Research has shown that healthcare practitioners have difficulty switching off from work, which impacts their well-being (Dures et al. 2010). Burnout and adverse psychological sequelae may result from accumulated stress and ineffective coping and support.

Very few studies focus on the perceptions of healthcare practitioners who care for patients and families with EB (Dures et al. 2010). Our study aimed to determine the perceptions, impact and needs of healthcare practitioners caring for patients and their families with EB, offer recommendations for the South African context and ultimately work towards improving health services for patients with EB.

## Research methods and design

### Study design

The study was based on qualitative, interpretative phenomenological analysis (IPA), a methodology focused on describing and interpreting lived experiences (Eatough & Smith 2017). Interpretative phenomenological analysis was a suitable design with the aim of understanding the experiences and perceptions of healthcare practitioners because IPA was based on interpreting and describing lived experiences and perceptions. Individual in-depth interviews were conducted to identify healthcare practitioners’ meaningful perceptions and experiences in caring for patients with EB.

### Setting

Individual interviews were conducted in quiet closed offices at two tertiary hospitals, Grey’s Hospital in Pietermaritzburg and King Edward VIII Hospital in Durban, respectively, removed from clinical settings to ensure privacy and confidentiality.

### Participant selection and sampling strategy

A non-probability, purposive, diversity sampling to saturation was used. Ten healthcare providers from various departments at the two specialist centres who had provided treatment and care for patients with EB during the study period were included in the study. Epidermolysis bullosa is a very rare disease, with both specialist centres seeing, on average 8 to 10 patients annually. All the selected participants had previously managed patients with EB, and staff that had not previously treated patients with EB were excluded from the study. The participants were members of staff that included a neonatologist, a paediatrician, a dermatologist, dermatology medical officers, dermatology-trained nurses, a paediatric palliative care nurse and a palliative care paediatrician. A fair representation of healthcare practitioners caring for EB patients in both specialist centres reached saturation with a sample size of 10 participants. Junior and senior staff were interviewed to represent diverse professional experiences.

### Data collection

Interviews were conducted by a postgraduate psychology student trained in the interviewing method and orientated to the EB context by the principal investigator (PI) A.V.C. and collaborator D.B., respectively. A set of questions was drawn up and was pilot tested in an initial interview (Appendix 1). Debriefing meetings between the interviewer and the PI A.V.C. took place after each interview for preliminary familiarisation with the interview content, impressions, emerging themes and reflexivity.

Interviews were digitally audio-recorded and transcribed verbatim by the interviewer. Information was processed and stored electronically using an appropriate data organising software package.

All participant data were de-identified and coded to ensure the confidentiality and anonymity of participants. Information was stored on the researcher’s private computer and password protected. Hard copies and electronic copies of data and voice recordings were planned to be destroyed 5 years after the completion of the study. The codes were retained separately from the data. All data were backed up to the Cloud^®^. Commitments to sharing study’s findings with individual participants were honoured.

### Data analysis

A qualitative systematic inductive analysis was used following Smith’s approach (Smith, Flowers & Larkin 2009). Each of the transcripts was read and re-read by the PI A.V.C. and the collaborators B.D., C.A., to obtain a general sense of the content and develop a table of themes. Annotations were made on the transcripts independently by the A.V.C., D.B. and C.A., and meaningful themes werecaptured and cross-referenced in the right-hand margin or review function of Microsoft^®^ Word. D.B. and PI A.V.C. each devised draft master tables of themes and theme clusters and then combined and compared them independently. Links between themes were highlighted. Themes were organised using thematic network diagrams devised by Attride-Sterling (2001) into three tiers tabulated as basic, organising, and global themes. A similar team-based data analysis has been used in other recent health research in the local context (Blackbeard & Aldous 2021; Dhada & Blackbeard 2019). During the write-up, a final integration was possible with phases of reflection and review among all four of the research team, following the IPA systematic inductive analysis, to describe and interpret the participants’ perceptions as accurately as possible. Sources of triangulation included the researcher’s reflective diary, correspondence among the research team, interview information sheets with the interviewer, and debriefing notes. These sources were used to familiarise and induce preliminary themes in a team-based process.

### Trustworthiness

Guba’s framework of trustworthiness, further refined by Shenton was used to ensure rigour (Lincoln & Guba 1986; Shenton 2004). The elements of credibility, transferability, dependability and confirmability were therefore used to evaluate trustworthiness.

Credibility was ensured through investigator triangulation, frequent debriefing sessions, member checking, and iterative questioning to ensure the correct interpretation of meaning. A thick description of the research findings ensured transferability. The PI A.V.C. did not interview the participants to avoid a dual role bias because the PI A.V.C. was a dermatologist specialising in EB and worked closely with the participants. The use of consensus among independent coders ensured dependability. Sources of triangulation included the primary investigator’s reflective diary, correspondence email and text among the research team, interview information sheets with the interviewer and debriefing notes to ensure confirmability.

### Ethical considerations

The significance and purpose of the study were explained to the healthcare practitioners before informed written consent was obtained. Information sheets and consent forms using non-technical language available in English and IsiZulu were given to each participant. Participants were ensured confidentiality and informed that they could voluntarily withdraw at any point in the study.

Approval was obtained from the health facilities and the KwaZulu-Natal Department of Health (KZ_202203_032). Final ethical approval for the study was then obtained from Biomedical Research Ethics Committee of the University of KwaZulu-Natal (BREC/0000768/2022).

## Results

### Sample characteristics

Ten healthcare practitioners were interviewed. They were from diverse ethnic and cultural backgrounds with varying expertise, experience and knowledge about EB (Table 1).

**TABLE 1:** Demographic profile of healthcare practitioners.

Respondent	Discipline	Rank	Number of years of practice	Number of EB treated	Demographics
1	Paediatric neonatologist	Consultant	15	2 per year	Indian female
2	Paediatric palliative care	Consultant	18	8	White female
3	Dermatology	Medical officer	1	2	African female
4	Dermatology	Consultant	6	< 10	African female
5	Paediatrics	Consultant	15	1 per year ± 15	Indian male
6	Dermatology	Enrolled nurse	9	7	African female
7	Dermatology	Enrolled nurse	9	7	African female
8	Dermatology	Senior medical officer	7	6 per year	African female
9	Paediatrics	Registered nurse	30	7	White female
10	Dermatology	Registered nurse	35	7	Indian female

EB, epidermolysis bullosa.

### Thematic integration

Six global themes were derived from the organising themes, each related primarily to perceptions or impact and needs. These were, in turn, clustered from meaningful basic themes. The themes were as follows:

1. *Understanding of the disease* (‘a diverse congenital skin condition requiring multimodal treatment and care, with guarded outcomes, sometimes poor based on the type and severity’).
2. *The emotions of parents, family and the healthcare practitioner* (‘The condition evokes emotional responses in parents, family, and healthcare practitioners’).
3. *Belief systems of the family and the impact on care* (‘African traditional beliefs about illness causation and traditional practices and expectations can constrain care and pressurise the mother’).
4. *Coping strategies of the healthcare practitioner and the team* (‘Coping is varied and a learning process, involving individual, group and team processes’).
5. *Helping parents and caregivers to cope* (‘An effective system with appropriate multimodal support and appropriate services will help parents and caregivers to cope and adjust’).
6. *Needs of the healthcare practitioner* (A need for continued medical education, research and multimodal biopsychosocial intervention).

#### Healthcare practitioners’ understanding of the disease process

Three organising themes were identified. These were perceptions of (1) pathogenesis; (2) treatment and (3) outcomes and prognosis of EB.

**Pathogenesis of the disease:** There was a common understanding among the doctors that EB was a genodermatosis with a variety of subtypes that determines the course of the disease. Two black African nurses shared that before caring for children with EB, they assumed this condition was because of cultural misfortune:

‘Before, I didn’t know anything I thought they were doing traditional things; that’s why the child ended up like this.’ (Respondent 6, enrolled nurse, female)

The respondents made associations, with one noticing a seasonal variation and the other noticing a geographical association with the number of patients seen.

**Views regarding the treatment and care of these patients:** There was unanimity that there was no cure for this condition and that treatment was aimed at counselling the family about the patient’s care, the importance of nutrition, preventing new lesions, pain management, complications and future pregnancies:

‘There is nothing, no treatment that we can give to undo the disease, so all of the disease management is really palliative in terms of symptom control, pain management, the dressings and counseling the families on how to care for that child with such fragile skin uhm and obviously the genetic component looking for future pregnancies uhm the risk of recurrence.’ (Respondent 2, consultant, female)

There was a concern that healthcare practitioners are not well informed about this condition and may be unable to manage these patients adequately:

‘I just think that caring for a child like this is difficult. I don’t think that a lot of health professionals really know much about it because it is quite rare uhm, so I am not convinced that it is always managed really well.’ (Respondent 2, consultant, female)

The respondents had varying views regarding the extent and level of care these children should receive. This variation appeared to be somewhat linked to the discipline of the healthcare practitioner. The perception in some disciplines was to go to any extent to ensure that the patient receives the same level of care as any other child without this rare disease. In contrast, other disciplines were more cautious and considered the resource limitations and the impact this disease had on the family and their finances.

There was also a sense of constraint and frustration with the limitations to care in a resource-limited environment. A paediatrician shared that one needs to be aware of the local environment, limited resources and the possibility of capping care when offering treatment to our patients:

‘I don’t think there’s much that can be done (.) dressings are expensive if you’re gonna treat it properly. In a first-world country, you see survival, but here in a second-world country, even with your best intentions, it just really doesn’t happen because it’s expensive, and it’s also just difficult for the parents; it changes their whole lifestyle.’ (Respondent 6, enrolled nurse, female)

**Views regarding the outcomes and prognosis of patients with epidermolysis bullosa:** Paediatricians highlighted that dermatologists and paediatricians work in ‘silos’ and that there was no centralised system for monitoring these patients. The paediatricians manage them at birth and then during complications and end-of-life:

‘We do not have a system to know what happens to these babies if they go to another hospital, we do not have that follow up, we don’t have a centralised system of monitoring these patients.’ (Respondent 1, consultant, female)

Prognostication of these patients is very difficult in a resource-limited environment, which resulted in tension between health professionals. A senior paediatrician shared that their perspective had changed once they were empowered about the condition. An improvement in care and outcomes of patients had also been helpful, although the overall prognosis remained poor.

There was agreement that the prognosis was extremely poor in the local setting, with a poor quality of life and risks of infections and sepsis. There was some shared despondency at the prognosis of this condition:

‘As a doctor also looking at this child that you know that uhm might not live for long because usually, like I’ve never seen an EB baby who is more than say two months, as a doctor you do everything that you need to do.’ (Respondent 4, consultant, female)

#### Impact

**Emotional impact:** Three organising themes were identified: the emotional impact on (1) the healthcare practitioner. (2) parents or other primary caregivers; and (3) family.

**The emotional impact on the healthcare practitioner treating a patient with epidermolysis bullosa:** Healthcare practitioners shared immense sadness, fear and distress during the consult and dressing changes. They shared that caring for patients with EB was distressing for both the parents and the HCPs. Seeing the patients in physical pain and the mothers in emotional pain was difficult.

One respondent shared feeling distressed and hopeless watching a child suffer from poor quality of life, being bullied into surviving, and knowing they will never be like other children:

‘They still couldn’t be normal children … they can’t do anything. I found that we’re bullying a child into a longer-term survival but the quality of life is still poor, so for me it’s a huge mixed bag of emotions.’ (Respondent 2, consultant, female)

Another respondent shared feeling overwhelmed by the circumstances of her patients, the sheer poverty, food insecurity, a lack of employment, families losing their homes and a lack of access to government subsidies. These challenges were perceived as increasing burnout.

Fear of the condition and the possibility of death was a significant concern among the nursing staff. They have shared concerns that the babies with EB be seen in the paediatric department and not in the dermatology clinic if their condition deteriorated during the consult:

‘I do a small prayer … God, please make sure that I do this dressing and I finish and this child leaves this department still breathing ‘cause you always check if they still breathing.’ (Respondent 7, enrolled nurse, female)

They felt pressured by the great expectations of the parents that their medical intervention would result in a cure. There was a sense of relief when the family had a faith-based or traditional belief system that gave hope for relief rather than the expectation of a cure placed upon the healthcare practitioner. One respondent shared a sense of relief when the child passed away because this meant an end to the suffering of these patients and their families:

‘Watching those parents getting worn down and worn down this poor child going through so much, I also feel quite relieved when the child dies to be honest.’ (Respondent 2, consultant, female)

Some found it difficult to separate emotions, remain unbiased, and not be led by the feelings of being a mother. Some disciplines felt sheer fear from not knowing what to do. Fear of the disease process and that handling the patient would result in further blistering and denudation of skin or not knowing how to manage the patient’s pain adequately. Healthcare practitioners felt a sense of failure that they could not do more. This could make the family feel abandoned as the healthcare practitioner was no longer focused on them.

Limited resources were perceived to impact how healthcare systems manage and the extent to which care is available for patients. This can have an impact on the healthcare practitioner as a moral injury:

‘When we know we can do more but don’t have the resources to do it, we have a bit of moral injury.’ (Respondent 5, consultant, female)

One respondent believed that palliation was humane and that not everything that can be treated should be treated as that would needlessly prolong suffering.

The respondent suggested that parents be empowered to be a part of their children’s decision-making and that the child’s best interest, whatever the prognosis, is pivotal to the care process. The care plan should be discussed with the parents and the child to hear and meet their needs. Parents should be given latitude regarding the care of their children without the healthcare practitioner being too prescriptive.

**Impression of the emotional impact on the parents or other primary caregivers caring for a child with epidermolysis bullosa:** The mother was usually observed to be the primary caregiver, with many being young, unmarried, and unsupported by their partners and their baby, with EB being their first child. The lack of support from their partners was noticed, either because of work or the emotional strain and withdrawal as a coping mechanism.

Parents’ emotions were described as being sad, distressed, confused, anxious, depressed, and in emotional turmoil about having a child with a painful, incurable disease. One respondent shared that she had observed dichotomous reactions from parents where some were hopeful and made personal adjustments to help their child, whereas others were angry and had even rejected the baby. She shared that once the parents lose hope, they physically and emotionally detach from the baby:

‘I think there is a lot of emotional turmoil that parents go through watching their child suffer, watching their child be in pain.’ (Respondent 1, consultant, female)

There appeared to be tremendous stress placed on the couple, sometimes leading to the ending of relationships. Couples and families blamed one another for the child having the genetic condition. The sick child could become a focal point in the family at the expense of family cohesion. There was also guilt because of the inheritance of the condition and because they felt that they might have performed something that led to the child’s suffering:

‘It is incredibly stressful for these parents, and some manage better than others; there have been marriages that have split up because the father couldn’t cope.’ (Respondent 3, medical officer, female)

The palliative care specialist had observed that many parents were relieved when their children died, relief that their children would no longer suffer. There were also feelings of immense devastation and sadness:

‘When the child does die of EB I often find that there’s uhm huge relief from the parents’ point of view obviously devastation and sadness but relief as a big thing.’ (Respondent 2, consultant, female)

African patients from a resource-limited background were found to have a greater level of acceptance than more resourced populations. The more affluent families were experienced as more demanding with high expectations from the healthcare practitioner. Acceptance was also attributed to cultural differences. All parents, regardless of economic or cultural differences, appeared to have similar emotions but different levels of acceptance.

**Impressions of the emotional impact on the family members and siblings of the index patient:** The HCP observed that having a sick child affects and impacts the entire family.

There are a lot of emotions and mixed reactions reported in siblings. They are fearful and cannot touch and play with their sibling in the traditional sense. There may be jealousy and isolation as attention is given to the baby:

‘The patient gets a lot of attention, so we even have a little bit of jealousy and uhm then guilt because they are jealous of their sick sibling, so that’s quite an interesting dynamic for the siblings.’ (Respondent 9, registered nurse, female)

It was noticed that families find it difficult to accept and make sense of this disease. They attribute the illness to jealousy and bewitchment by other families, a belief that they have not prayed enough, and the anger of the ancestors for displeasing behaviour or a lack of rituals that should have been performed in the family.

**Impact on care – Healthcare practitioners’ perception of belief systems of the family and the impact on care:** Several respondents noticed that patients went to traditional healers before or after consulting allopathic healthcare facilities. Cultural belief systems were seen to play an essential role in the understanding of the disease. The respondents observed that African belief systems ascribe disease aetiology to the ancestors’ anger or witchcraft. They felt the parents did not understand the disease process and thus turned to cultural belief practices:

‘It’s difficult to them … they cry, they don’t understand [*and*] they think the ancestors are angry with them, that’s why the child ended up like this.’ (Respondent 6, enrolled nurse, female)

One respondent noticed that allopathic healthcare was disease-focused, and it was not routine practice for health professionals to enquire about traditional cultural or religious beliefs with their patients despite this being essential for holistic care.

The respondents had differing views regarding the family consulting a traditional healthcare practitioner (THP). The majority supported the family seeking consultation, whereas others thought it would bring about more confusion, was dangerous, and the THP consult fees could be put to better use in caring for the baby.

However, all shared concerns that the practices such as rubbing substances and scarification on the already compromised skin of patients with EB could lead to infection. The ingestion or the use of enemas might have systemic effects as these medications have not been scientifically tested and validated. The respondents acknowledged that the family might find relief from THPs in bringing about closure and the reassurance that they have performed all that they could for their child without regret:

‘I wouldn’t say they mustn’t go because they go there because they want to get closure but they also need to understand that not everything is about Sangomas. They must be conscious because whatever they bring from that Sangoma might cause infection to this child especially if they are going to apply it on the baby, also with these cuts that they do.’ (Respondent 7, enrolled nurse, female)

Some respondents emphasised the importance and benefit of holistic care and engaging with the family about their belief systems in a non-judgemental manner. This was perceived to encourage honest dialogue and set the stage for the education of safe healthcare practices within traditional practices. One respondent suggested that traditional healers be included in the allopathic healthcare system.

There was an overall impression that African traditional beliefs about illness causation and traditional practices and expectations could constrain care and pressure the mother.

#### Needs and coping strategies

**Needs of the healthcare practitioner:** Healthcare professionals observed that empowering obstetricians, midwives, paediatric staff, and staff at the peripheral care centres to identify the condition, training in basic wound care, and promptly referring these patients to a specialist centre was essential. This would prevent further blister formation, detect and treat complications such as infections timeously, and assist significantly in caring for these patients and their families.

They expressed a need for training in genetics counselling to educate and empower families to make informed decisions about future pregnancies. There was also a need for training in palliative care to break distressing news to families and manage pain effectively:

‘I don’t feel confident in managing pain … I am afraid to use morphine in babies.’ (Respondent 4, consultant, female)

A respondent shared that more research was needed on this rare disease, and she wished a cure could be found.

They shared a need for multimodal biopsychosocial intervention with access to psychology and assisted programmes to assist with coping mechanisms:

‘I feel defeated when a baby dies, we need counseling.’ (Respondent 8, senior medical officer, female)

**HCP perceived needs of the parents:** Three organising themes were identified:

**System (collaborative continuity with specialised support, timed referrals, and capacity needed to manage best):** A dedicated centre of specialised care was recommended to be essential for these families. A respondent noticed that the healthcare system was fragmented in that a patient sometimes saw a different practitioner at each visit, which made building relationships and continuity near impossible:

‘The way our system is structured is very fragmented, so you either getting care in hospital but once you leave hospital you generally don’t see that team again, you come to outpatients and you might see someone different every time.’ (Respondent 1, consultant, female)

It was also emotionally taxing for the mother to explain her child’s condition to every healthcare practitioner who, in most cases, had never seen this condition before and then subjected them to shock and horror.

It was suggested that because traditional health practitioners play a big role in our community, it would be best to incorporate them into the healthcare system so they too can be empowered as they were the primary care providers for many patients.

They suggested that it would be ideal for a multidisciplinary team composed of dermatologists, medical officers, paediatricians, neonatologists, palliative care specialists, psychologists, social workers and wound care specialists.

**Support (counselling and support to overcome emotional barriers and have appropriate skills, expectations and hopes):** Extensive counselling, education and empowering parents were found to help them adjust and adapt to their child’s condition. Relieving them of the burden that this condition was not their fault and that there was nothing that they could have carried out to prevent this from occurring can be a huge weight lifted from these parents, especially when cultural beliefs were perceived to be judgmental of behaviours or omissions which resulted in their child having this condition:

‘We need to spend time with parents to explain the condition so that they will know that it is not their fault.’ (Respondent 8, senior medical officer, female)

It was highlighted that family support was essential to help shoulder the disease’s emotional and physical burden, such as assisting with lengthy dressing changes. Consistency for a mother and the family was also important. A process of learning and acceptance was described, relieving worry, uncertainty, emotional turmoil or a lack of support.

**Services (need for specialised services, palliative care, psychological and psychosocial support):** The paediatricians noticed that with the improvement in diagnostics and, therefore, prognostication, limited resources would be used appropriately to help patients that should have a better outcome. Introducing parents to psychologists and the palliative care team early in the child’s illness was suggested to be more beneficial than a late referral. The palliative care team is available to the family, providing support and a safety net, walking the road with them, and in severe cases, preparing them for the possible death of their child.

It was emphasised that the mothers and family receive continuous medical education, emotional and physical support, and empathy to encourage family unity and referral to allied health services for psychosocial support and psychological interventions:

‘There will be stages for the mother … denial, anger[and]all of the usual grief stages helping deal with that uh would also be on the psychology sometimes social worker side depending on whatever is necessary, and then the skills in terms of mom to learn how to do dressings.’ (Respondent 9, registered nurse, female)

Overall, an effective system with appropriate multimodal support and appropriate services would help parents and caregivers cope and adjust.

#### Coping strategies

**Coping strategies of healthcare practitioners treating patients with epidermolysis bullosa:** Various coping strategies were described. Some felt that healthcare practitioners were vulnerable and did not have good coping strategies.

Some respondents said they coped by being clinical and relying on evidence-based medicine alone. A palliative care nurse shared that she can compartmentalise her emotions and manage well with being there for a dying child and their family. She considered her stoicism as a God-given gift. She commented that her colleagues battled with seeing children die. Others noticed that seeking psychosocial support, the help of a psychologist, turning to religion and spirituality, debriefing with the team or a close family member, practising mindfulness, yoga, or other methods, or physical exercise such as running.

Some defensive coping skills employed by respondents included suppressing emotions not to upset the mother, swapping out duties, or making an excuse to leave the unit during dressing changes to avoid emotional trauma:

‘I think it’s better if I knew before if there is an EB coming, cause I have to do self-counseling, cause it’s not easy. It breaks my heart honestly speaking the first two or three I was dodging them. I will just leave and say I have something to do so that I won’t do it.’ (Respondent 7, enrolled nurse, female)

Other respondents reported less effective coping strategies or no strategy at all such as stress eating, difficulty separating roles as a doctor and mother, and as a result, breaking down and crying when confronted with difficult decisions around the care of an EB baby, or removing themselves when an EB baby entered the department.

A respondent noticed that HCP varied in their coping strategies, some with far-reaching adverse outcomes:

‘I think each individual doctor learns their own way of coping, uh so some are successful and some are not high rate of the substance abuse and uh you know even suicide among doctors, certain professions or certain disciplines more.’ (Respondent 5, consultant, female)

**Coping strategies of the team treating patients with epidermolysis bullosa:** Departmental or team coping strategies varied among disciplines. Staff in some disciplines felt that being equipped with knowledge about EB, debriefing sessions, checking in with each other, feedback sessions and support from seniors assisted them to cope. In contrast, the staff in other departments felt less supported. A junior doctor noted that the senior doctors get very attached to EB patients and the family, and it was their opinion that this leads to dependence and a false sense of hope.

In general, team self-care, particularly debriefing, meetings, burnout prevention and empowered team culture, was perceived to assist with coping.

## Discussion

Treating a rare and incurable genodermatosis such as EB can take its toll on a healthcare practitioner caring for a child and their family with this disease. Diagnosing EB subtypes is essential to prognosticate, counsel families, and plan therapy. This is challenging in a resource-limited environment such as South Africa. The diagnosis of the exact subtype is mainly made clinically with specialised tests such as electron microscopy needing to be sent to specialist centres hundreds of kilometres away; genetic testing is limited to research laboratories and private facilities, and immunofluorescent mapping, which is the gold standard, is not offered in South Africa. There are only two paediatric dermatologists in South Africa with specialist EB units in KwaZulu-Natal and Cape Town. Procuring dressings can be a challenge even in these centres.

Healthcare practitioners knew that there was no cure for the condition, outcomes were guarded, and management is supportive and should be holistic and biopsychosocial, considering the impact the disease has on the whole family. There was a concern that health professionals outside of a specialist centre were unfamiliar with the condition and the precautions that needed to be taken when managing these patients and their families. This was a similar concern of parents in a study by Yuen et al. (2012).

There were divergent views among THPs concerning the extent of care, coping strategies, and the incorporation of traditional health professionals in allopathic healthcare.

The respondents differed in the treatment of patients with EB. Some felt that all efforts must be taken in treating patients with EB. Others thought one needed to be circumspect and consider the resource limitations and the impact of the disease on the family. Resource limitations impacted management decision-making resulting in moral injury to the healthcare practitioner. A healthcare practitioner expressed concern that the medical fraternity works in silos with a lack of comprehensive centralised centres of care. Treating a patient with a rare disease with no cure can pose an ethical dilemma for the healthcare practitioner, grappling with the decision to treat or not to treat (Boesen et al. 2016). Yan et al. (2007) suggested that patients with a severe form of EB should be offered palliation with no aggressive management.

Healthcare professionals have shared feelings of being overwhelmed, afraid, distressed, fearful, sad and hopeless. A nurse shared feeling terrified that the patient could die during the consult and excused herself when EB patients were in the unit. Some respondents reported feeling pressure from parents to cure their children, and some felt great relief at the passing of the patient and the end of suffering of the patient. Dures et al. (2010) highlighted that healthcare practitioners feel frustrated at the limited effects of treatment and feelings of guilt, powerlessness, and inadequacy, knowing there is no cure for EB and that the easing of symptoms was transitory. They had difficulty switching off, having journeyed with the family over the years, and feeling frustrated and self-critical when they could not change the trajectory of the disease process. There were no boundaries to care, where healthcare practitioners offered support beyond their expertise, such as being a counsellor and confidant (Dures et al. 2010).

The respondents found that the mothers were the primary caregivers, were from a poor economic background, were young, unmarried, and had little support from their partners. Mothers displayed a wide range of emotions, from anxiety, confusion, guilt, shock, depression and hopelessness to being physically and emotionally detached (Von der Lippe, Diesen & Feragen 2017). Fathers were mainly absent, and those who attended the consult appeared emotionally and physically detached. Our observations are similar to a study by Chogani et al. (2021), who observed that mothers were the primary caregivers, were from a poor economic background, and were more vulnerable, affecting parents’ quality of life, thus requiring special attention. Cardinali, Migliorini and Rania (2019) documented the experiences and challenges of fathers with a child with a rare disease. The respondents observed that the patient’s condition negatively impacted the parents’ personal relationships. Discord was also observed between the parents, blaming each other for the inheritance of the condition. Fine et al. (2005) observed that EB impacted a couple’s relationship, on parents’ decision to not have more children, with 22% ending in divorce. A palliative health professional noticed that some parents were relieved at the death of their children, marking the end of suffering coupled with deep loss and sadness. In a study by Yuen et al. (2012) in the Netherlands, where euthanasia is permissible, parents expressed a need to be part of planning the end of life. Parents are often blamed by the extended family for the inheritance of the child’s condition. This was similar to the findings in the Chinese community in which the mother was blamed for the inheritance of the disease (Wu et al. 2020). They observed that there was an impact on the patients’ siblings as the focus was placed on the patient leaving the siblings feeling afraid, isolated and jealous. These findings were similar to other studies in the literature (Hilkner et al. 2019; Plumridge et al. 2011; Yuen et al. 2012). Siblings have been described as the ‘glass child’, invisible in the face of their sick sibling (Hanvey, Malovic & Ntontis 2022).

There were concerns that traditional practices were unscientific and may be harmful, similar to views found in the literature (Courtright 2000; Hopa, Simbayi & Du Toit 1998). It was found that cultural belief systems impact the parents’ understanding of the disease, compliance with care, and acceptance of the condition. It is, therefore, essential to address cultural and religious belief systems for the holistic care of patients. Touch is a crucial sense that forges bonds between people; however, this vital sense can harm a patient with EB. Carrying a baby on the back is deeply entrenched in the African culture; it is a form of comfort and nurturing to the infant and allows the mom to perform household chores while caring for her infant (Zaidman-Mograbi, Liana Le & Hall 2020). This would be near impossible in caring for a baby with EB. In the African culture, a baby is introduced to the family; thus, limiting touch could have a far-reaching negative effect. Healthcare practitioners shared that families also attributed illness to the ancestors’ anger, bewitchment, or the lack of performing vital rituals. These beliefs are similar to the findings of Shizha and Charema (2012).

The respondents emphasised the need to support, educate and counsel families and dispel traditional and family myths that assign blame to the parents. Family support and working in a multidisciplinary team are essential for holistic care and to empower the parents and the patient to be part of the decision-making process and therapy.

According to the respondents there is a need for further research on this rare disease, more accessible and precise diagnostics to aid prognostication, and healthcare practitioners continued medical education to identify a baby with EB and promptly refer them to specialist centres (Martin et al. 2019). There was a perceived need for more dedicated specialist centres to ensure rapid diagnosis, care and consistency for patients and their families. There appeared to be some feeling that the role of the THP should be acknowledged as they might be the first care contact for the patient. Healthcare practitioners expressed a need for palliative care and genetics counselling training to manage the patient and their families comprehensively.

Coping strategies varied among the respondents, possibly reflecting differences between disciplines and units. Some respondents felt supported and employed various healthy coping strategies, whereas others felt disempowered and overwhelmed, using avoidance techniques as a protective mechanism. They suggested that referral to psychology or assisted programmes were needed to support healthcare practitioners dealing with EB care (Martin et al. 2019).

### Implications or recommendations

The following recommendations are tentative and based on the findings of this research.

There is a need for rare diseases such as EB to be highlighted to government and policymakers and for resources to be made available for research, diagnostics, training of health professionals, and improved treatment for the holistic care of these patients and their families.

Healthcare practitioners partnering with advocacy organisations such as Rare Diseases South Africa and DEBRA South Africa (Dystrophic Epidermolysis Bullosa Research Association) by forming family support groups within various hospitals and collaborating in research may be a great benefit for patient care.

There is a need for specialist centres to ensure continuity of care for the patient and their families, a multidisciplinary approach with early involvement of the palliative care team, allied health practitioners, and genetic counselling so families can make informed decisions.

Empower parents with written material and practical workshops on EB. Fathers tend to be less affected as the mother is the primary caregiver. Therefore, they need to be counselled and empowered to support their partners and children.

There is a need for multidisciplinary meetings and continuing medical education to empower healthcare practitioners and allied health practitioners who care for these patients and their families. This could include outreach programmes at various health institutions or online Zoom^®^ or Teams^®^ meetings. There is a need for clinical practice guidelines for patients with EB in a resource-limited environment such as South Africa. These guidelines aim to empower all healthcare practitioners at primary, district, regional and tertiary healthcare facilities.

Healthcare practitioners run the risk of burnout, always being available with little downtime. There is a need for healthcare practitioners to have debriefing sessions and possible referrals to employee-assisted programmes or psychologists at their hospitals to acquire healthy coping skills. There is a necessity for wellness programmes within hospitals and clinics that empower staff and focus on self-care.

It is essential to address the cultural belief systems of the patient as this may impact the care of the baby. Creating an open dialogue in a non-judgemental manner is vital to care. Discussing their belief system with the family is very important as meeting them at their point of need.

### Limitations

Given that the study was small-scale and based upon non-probability sampling, the findings and their generalisability should be considered tentative and exploratory. The results invite further empirical study of the impact of caring for EB on healthcare practitioners.

Further research may focus on the impact on healthcare professionals in all the disciplines that manage patients with EB and their families to determine if there is an interdisciplinary difference in the impact, coping skills, and needs of these healthcare professionals.

## Conclusion

Caring for patients and their families with EB can be rewarding and emotionally taxing for the healthcare practitioner. Healthcare practitioners wear various hats, being the healthcare provider, the counsellor, the confidant, and the patient’s advocate by lobbying for better care for their patients. Healthcare practitioners not only experience the uncertainty and guarded prognosis of the disease as stressful but also witness the emotional impact on the parents and family of the patients, sharing a sense of helplessness and diminished hope. Effective systems of care and multimodal support are crucial to assist parents and caregivers in adjusting and coping. Awareness of work’s impact on the healthcare practitioner and seeking healthy coping strategies are essential for a healthy life. It is also crucial that healthcare practitioners realise their needs and limitations and seek the assistance of support groups and allied health professionals. Healthcare practitioners should be cognisant of self-care, healthy coping strategies, a need for time out, and the proverbial reminder that ‘we cannot give from an empty cup’.

## Figures and Tables

**FIGURE 1-A1:**
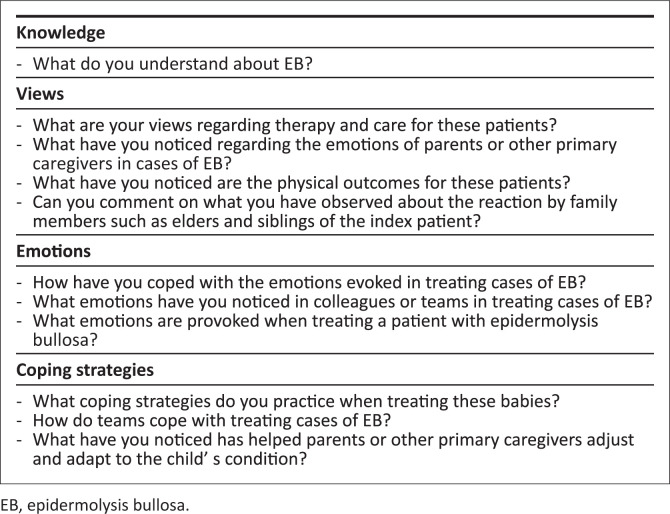
Semi-structured interview schedule.
